# The renal consequences of maternal obesity in offspring are overwhelmed by postnatal high fat diet

**DOI:** 10.1371/journal.pone.0172644

**Published:** 2017-02-22

**Authors:** Sarah J. Glastras, Hui Chen, Michael Tsang, Rachel Teh, Rachel T. McGrath, Amgad Zaky, Jason Chen, Muh Geot Wong, Carol A. Pollock, Sonia Saad

**Affiliations:** 1 Department of Medicine, Kolling Institute, University of Sydney, Sydney, Australia; 2 Department of Diabetes, Endocrinology and Metabolism, Royal North Shore Hospital, St Leonards, NSW, Australia; 3 School of Life Sciences, Faculty of Science, University of Technology Sydney, Sydney, Australia; 4 Department of Anatomical Pathology, Royal North Shore Hospital, St Leonards, NSW, Australia; University of Louisville, UNITED STATES

## Abstract

**Aims/Hypothesis:**

Developmental programming induced by maternal obesity influences the development of chronic disease in offspring. In the present study, we aimed to determine whether maternal obesity exaggerates obesity-related kidney disease.

**Methods:**

Female C57BL/6 mice were fed high-fat diet (HFD) for six weeks prior to mating, during gestation and lactation. Male offspring were weaned to normal chow or HFD. At postnatal Week 8, HFD-fed offspring were administered one dose streptozotocin (STZ, 100 mg/kg i.p.) or vehicle control. Metabolic parameters and renal functional and structural changes were observed at postnatal Week 32.

**Results:**

HFD-fed offspring had increased adiposity, glucose intolerance and hyperlipidaemia, associated with increased albuminuria and serum creatinine levels. Their kidneys displayed structural changes with increased levels of fibrotic, inflammatory and oxidative stress markers. STZ administration did not potentiate the renal effects of HFD. Though maternal obesity had a sustained effect on serum creatinine and oxidative stress markers in lean offspring, the renal consequences of maternal obesity were overwhelmed by the powerful effect of diet-induced obesity.

**Conclusion:**

Maternal obesity portends significant risks for metabolic and renal health in adult offspring. However, diet-induced obesity is an overwhelming and potent stimulus for the development of CKD that is not potentiated by maternal obesity.

## Introduction

Obesity is a known independent risk factor for the development and progression of chronic kidney disease (CKD) [1, 2]. Additionally, obesity is strongly associated with the development of type 2 diabetes (T2D), which in turn accounts for the majority of CKD in most countries worldwide [3]. As such, globally the incidence of CKD has been increasing in the setting of rising rates of obesity. Identification of individuals with a predisposition to the development of CKD may enable targeted and early intervention in order to prevent progression of kidney damage and reduce complications of CKD including cardiovascular disease and future end-stage kidney disease.

Maternal obesity has sustained effects on the risk of chronic disease in offspring. Evidence from both human and animal studies suggests that maternal obesity ‘programs’ the offspring towards obesity, dysglycaemia, diabetes and hypertension, all key features of the metabolic syndrome [4, 5]. This observation evokes the concept of the developmental origins of health and disease, which suggests that foetal exposure to factors inherent to the maternal milieu during gestation may influence programming towards chronic disease [6]. There is substantial evidence that maternal obesity increases the risk of metabolic-related complications including cardiovascular disease [7–10]. The effect of maternal obesity on the risk of CKD in offspring is less well appreciated though a large population-based study of young adults with CKD found a disproportionate number of children were born to mothers who were overweight or obese during their gestation [11].

Animal models of obesity are important to enable better understanding of the pathophysiologic pathways specific to obesity-related kidney disease. Our studies have previously demonstrated that diet-induced obesity in rodents can be utilised to study the developmental programming effects of maternal obesity on offspring’s kidney health [12, 13]. Most recently, we found that offspring of obese mothers had increased renal fibrosis, inflammation and oxidative stress which persist into adulthood at postnatal Week 32 [14]. When given an additional insult of streptozotocin (STZ, 55 mg/kg/day for 5 consecutive days) to induce diabetes, offspring exposed to maternal obesity had increased susceptibility to renal damage with exaggerated renal inflammation and oxidative stress [14]. However, induction of diabetes with STZ in this fashion is analogous to a model of type 1 diabetes with marked insulin depletion from pancreatic beta cells. Others have reported that one dose of STZ with HFD results in metabolic features of T2D and may induce CKD [15].

We hypothesised that maternal obesity may also augment the effect of diet-induced obesity-related renal damage especially when combined with one dose of STZ. Therefore, the aim of this study was to determine, using a C57BL/6 mouse model, whether maternal obesity exacerbates renal damage in HFD-induced obese offspring, specifically related to known mechanisms involved in obesity-related kidney disease including renal fibrosis, inflammation and oxidative stress.

## Research design and methods

### Animal experiments

The animal model of maternal obesity employed in this study has been previously described [14]. In short, C57BL/6 female mice were fed HFD or normal chow diet for six weeks prior to mating, during gestation and lactation. Male offspring were weaned to either normal chow or HFD at postnatal Day 20. At postnatal Week 8, offspring fed HFD were assigned to either high-dose STZ (100 mg/kg, intraperitoneal injection (ip), once) or vehicle control (citrate buffer). The model yielded six groups: CC: offspring of lean mothers fed a chow diet post-weaning, HC: offspring of obese mothers fed a chow diet post-weaning, CH: offspring of lean mother fed a HFD post-weaning, HH: offspring of obese mothers fed a HFD post-weaning, CH-STZ: offspring of lean mothers fed a HFD post-weaning together with one dose of STZ at postnatal Week 8, and HH-STZ: offspring of obese mothers fed a HFD post-weaning together with one dose of STZ.

This study was approved by the Royal North Shore Hospital’s Animal Ethics Committee (1309-007A) and complied with the Australian Code of Practice for the Care and Use of Animals for Scientific Purposes. All animals were housed in the Kearns Facility of the Kolling Institute and maintained at 22±1°C with a 12/12-hour light–dark cycle. They were monitored at least once per fortnight. Animal health, body condition and wellbeing were assessed each time. No adverse events occurred during any of the experiments described.

Intraperitoneal glucose tolerance tests (IPGTTs) were performed in fully conscious animals at Weeks 14, 20 and 30 after 6 h of fasting, as previously described [14]. Mice were placed in metabolic cages and a 24-h urine collection was performed one week prior to sacrifice. Euthanasia was performed by deep anaesthesia with isoflurane (4%) followed by cardiac puncture. Tissue harvesting took place at Week 32 under fasting conditions. Organ perfusion was performed with PBS after cardiac puncture for blood collection. The kidneys, liver, and fat were collected and weighed then the kidney was fixed in formalin or snap frozen in liquid nitrogen.

### Serum measurements

Serum insulin was measured using an ELISA method (Merck, Darmstadt, Germany). Serum total cholesterol, triglycerides and LDL was measured using the Architect C16000 Clinical Chemistry Analyser (Abbott Laboratories, Ill, USA). Non-esterified fatty acids (NEFA) were measured using a WAKO kit (Osaka, Japan).

### Analysis of renal function

Urine albumin and creatinine concentrations were determined using the Murine Microalbuminuria ELISA kit (Exocell, Philadelphia, USA) and Microcreatinuria ELISA kit (Exocell). Serum creatinine was measured using the Architect C16000 Clinical Chemistry Analyser.

### Analysis of renal structural changes

Paraffin-embedded kidney sections were stained with Periodic Acid Schiff (PAS). The whole kidney cortex was examined under the magnification of 400x using a light microscope (Olympus photomicroscope linked to a DFC 480 digital camera). Two independent assessors, an anatomical pathologist and nephrologist, reviewed histological sections in a blinded manner and scored tubulointerstitial fibrosis, tubular injury and glomerulosclerosis. There was high inter- and intra-observer agreement for histological analysis between observers; and an average of 20 individual scores was calculated to generate the overall glomerulosclerosis score.

The characteristic features of tubulointerstitial fibrosis include tubular atrophy or dilatation, presence of mononuclear inflammatory cells, widening of interstitial spaces with deposition of extracellular matrix, interstitial cell proliferation and wrinkling or thickened tubular basement membrane (perivascular and periglomerular areas were discounted). Tubular interstitial fibrosis was scored as described in S1 Fig. Further analyses of tubular injury were assessed by: (A) tubular dilatation, (B) tubular vacuolation, C) glycogenated nuclei, and (D) tubular casts and scoring was performed as described in S2 Fig. Glomerulosclerosis was scored as previously described [12, 16] and detailed in S3 Fig.

### Immunohistochemistry and semi-quantification

Paraffin-embedded kidney sections were deparaffinised and incubated with primary antibodies against collagen IV (dilution 1:1000, Abcam Ltd, Cambridge, USA), fibronectin (dilution 1:500, Abcam), or 8-hydroxy-2'–deoxyguanosine (8-OHdg) (dilution 1:200, Cell Signaling Technology, Beverly, USA) at 4°C overnight, followed by horseradish peroxidase anti-rabbit Envision system (Dako Tokyo, Japan) the following day.

Alternatively, frozen sections were incubated in the widely used markers of murine macrophage populations, rat anti-mouse F4/80 monoclonal antibody (dilution 1:100, ABD Serotec, USA) or rat anti-mouse CD68 antibody (dilution 1:100, ABD Serotec) for one hour. Thereafter, they were incubated with a secondary HRP labelled goat anti-rat antibody for 30 min (ABD Serotec, dilution 1:200).

Antigen-antibody reactions were visualized with 3.3diaminobenzidine tetrahydrochloride (Dako) and counterstained. The tissue specimens were examined by light microscopy using the Olympus photomicroscope. For fibronectin, collagen IV, 8-OHdg, CD68, and F4/80, six consecutive non-overlapping fields from each section of renal cortex were photographed under high magnification. Stained areas were quantified using Image J software (NIH, UK).

### RT PCR

RNA was extracted and RT-PCR was performed as previously described [14]. PCR primers are previously published [14], or are available from the authors upon request. The results are presented as fold change compared to control after normalisation to β-actin.

### Statistical methods

Results are expressed as mean ± standard error of the mean (SEM). Data were analysed using analysis of variance (ANOVA), and post-hoc Bonferroni tests to make between-group comparisons. To compare plasma glucose levels during the IPGTT, a two-way ANOVA was performed and between-group differences were determined using Tukey’s post hoc test. Analyses were carried out using GraphPad Prism 6.0 (GraphPad Software, San Diego, USA) and P value < 0.05 was considered statistically significant.

## Results

### Maternal obesity increases adiposity in the presence of obesity and STZ

**Fig 1:**
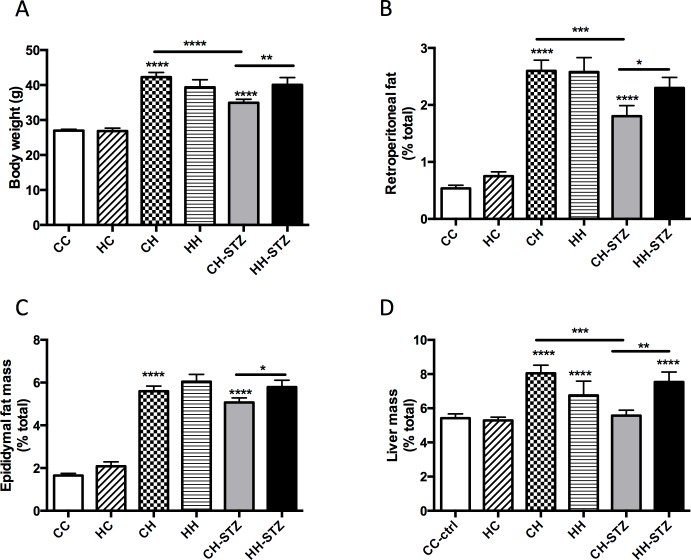
Anthropometric measures performed at Week 32. A. Body weight, B. Retroperitoneal fat mass relative to body weight, C. Epididymal fat mass relative to body weight, and D. Liver mass relative to body weight. Results are expressed as mean ± SEM, N = 9–20. *P< 0.05, **P<0.01, ***P<0.001, ****P<0.0001 compared to CC. Control: CC, offspring from obese mothers fed chow: HC, offspring of lean mothers fed high fat diet (HFD): CH, offspring of obese mothers fed HFD: HH, offspring of lean mothers fed HFD and administered one dose of streptozotocin (STZ) at Week 8:CH-STZ, and offspring of obese mothers fed HFD and administered one dose of STZ: HH-STZ.

As expected, animals fed HFD from weaning until Week 32 had increased body weight compared to control (P < 0.0001 for all groups vs. CC; Fig 1A). There was no difference in body weight of offspring of obese mothers weaned to a normal chow diet compared to control. Maternal obesity was associated with increased body weight in offspring fed HFD and given a further diabetes-related insult with STZ (CH-STZ vs. HH-STZ, P < 0.01).

Visceral adiposity, as measured by retroperitoneal and epididymal fat mass, was increased in all groups fed HFD (P < 0.0001; Fig 1B and 1C). Similar to the observation for body weight, offspring of obese mothers fed HFD and given one dose of STZ had significantly increased adiposity as measured by both fat depots compared to similar offspring of lean mothers (CH-STZ vs. HH-STZ, P < 0.05).

All groups fed HFD demonstrated hepatomegaly except for the CH-STZ. Though there was no effect of maternal obesity on liver mass in offspring fed HFD, offspring fed HFD and given STZ had increased liver size (CH-STZ vs. HH-STZ, P < 0.01, Fig 1D). Offspring fed normal chow diet had normal liver mass regardless of maternal diet.

### Maternal obesity worsens glucose intolerance in HFD-fed offspring

**Fig 2:**
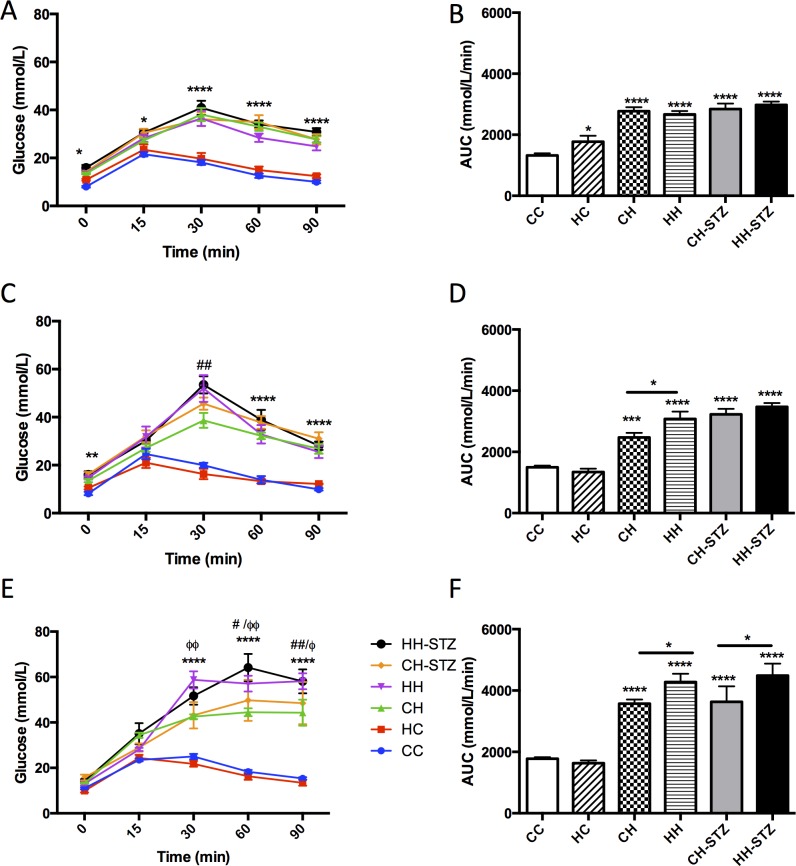
Intraperitoneal glucose tolerance tests performed at postnatal Week 14, 20 and 31 in C57Bl/6J mice. Blood glucose levels were measured at 0, 15, 30, 60 and 90 minutes post-glucose injection (A-C). AUC of A-C (D-F). Results are expressed as mean ± SEM, n = 8–13. *P< 0.05, **P<0.01, ***P<0.001, ****P<0.0001 compared to CC.

IPGTTs were performed at Week 14, 20 and 30, which corresponded to 6 weeks, 12 weeks and 22 weeks post-STZ induction of diabetes respectively (Fig 2). The between-group differences of relevance included the effect of HFD and maternal obesity on glucose tolerance.

Week 14: The HFD-fed groups had significant fasting hyperglycaemia compared to control at Time 0 (CH vs. CC, P < 0.05, CH-STZ vs. CC, P < 0.05, HH vs. CC, P < 0.05 and HH-STZ vs. CC, P < 0.01, Fig 2A). There was no effect of maternal obesity on fasting glucose levels regardless of the postnatal diet. At each timepoint, hyperglycaemia was sustained in the HFD-fed groups (Times 30, 60 and 90: each HFD-group vs. CC, P < 0.0001, Fig 2A). There was no added effect of maternal obesity in the HFD-fed groups on blood glucose values at any time. In all HFD-fed groups the AUC was increased (CH vs. CC, CH-STZ vs. CC, HH vs. CC and HH-STZ, P < 0.0001, Fig 2B). Compared to offspring born to lean mothers, offspring of obese mothers weaned to normal diet had impaired glucose tolerance as measured by AUC (HC vs. CC, P < 0.05).

Week 20: At commencement of the IPGTT only the HFD-fed groups given STZ had significant fasting hyperglycaemia compared to control (P < 0.05, Fig 2C). From 30 minutes onwards, all HFD-fed groups had higher blood glucose levels compared to control (P < 0.0001). The AUC was increased in all groups fed HFD (P < 0.0001, Fig 2D). Maternal obesity exacerbated glucose intolerance in the HFD fed group (HH vs. CH, P < 0.05).

Week 30: At commencement of the IPGTT, there was no difference in fasting blood glucose levels between groups. By 15 minutes, some HFD-fed groups had higher blood glucose levels compared to control (CH vs. CC, P < 0.05, HH-STZ vs. CC, P < 0.001, Fig 2E). By 30 minutes, all HFD-fed groups had higher blood glucose levels compared to control, which were sustained until 90 minutes (P < 0.0001). Furthermore, maternal obesity potentiated the effect of HFD on blood glucose levels at 30 minutes (HH vs. CH, P < 0.01). At 60 minutes, maternal obesity potentiated the effect of HFD with or without STZ (HH vs. CH, P < 0.05, HH-STZ vs. CH-STZ, P < 0.01). Finally, by 90 minutes, hyperglycaemia persisted in all HFD-fed groups (P < 0.0001) and maternal obesity continued to exacerbate the effect of HFD with or without STZ (HH vs. CH, P < 0.01, HH-STZ vs. CH-STZ, P < 0.05). The glucose intolerance measured by AUC resulting from HFD was sustained until Week 30 (P < 0.0001, Fig 2F). Although maternal obesity alone worsened glucose intolerance at Week 14, by Week 20 the effect was lost.

### Maternal obesity has modest effect on metabolic measures in the setting of obesity and diabetes

**Fig 3:**
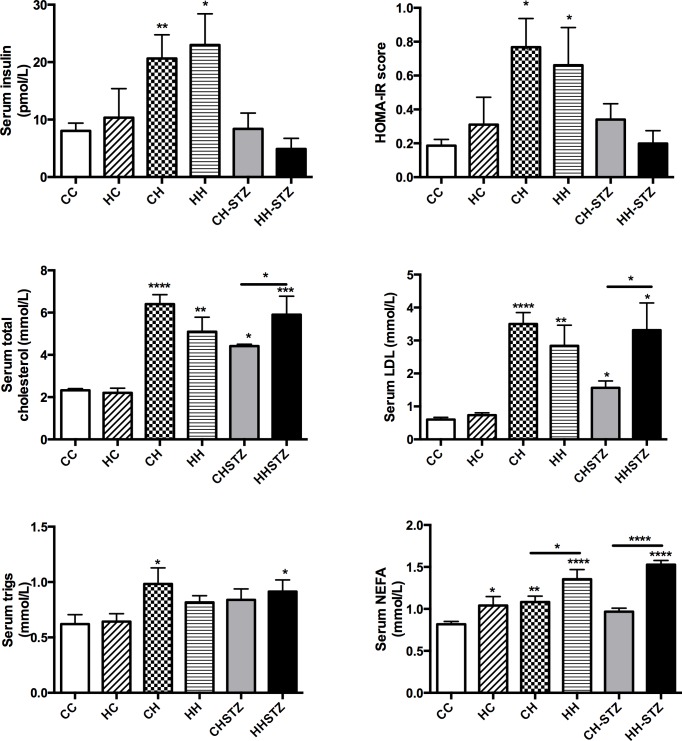
Metabolic measures at Week 32. A. Serum insulin, B. HOMA-IR score, C. Serum total cholesterol, D. Serum triglyceride measure, E. LDL concentration, F. NEFA concentration. Results are expressed as mean ± SEM, N = 6–10. *P< 0.05, **P<0.01, ***P<0.001, ****P<0.0001 compared to CC.

At Week 32, fasting hyperinsulinaemia was observed in HFD-fed offspring (CH vs. CC, P < 0.01, HH vs. CC, P < 0.05, Fig 3A). There was no differential effect of maternal obesity on insulin concentrations, regardless of group. Insulin resistance was measured by calculating HOMA-IR; there was evidence of insulin resistance in some HFD-fed offspring (CH vs. CC, P < 0.05, HH vs. CC, P < 0.05, Fig 3B). Offspring given STZ did not demonstrate insulin resistance despite their higher weight, adiposity and hyperglycaemia and maternal obesity did not exacerbate insulin resistance in adulthood.

Hyperlipidaemia, including total cholesterol and LDL, was evident in all HFD-fed offspring (Fig 3C and 3D). Maternal obesity potentiated hyperlipidaemia as measured by total cholesterol and LDL in HFD-fed offspring given STZ (P < 0.05). Serum triglyceride levels were increased in two of the HFD-fed offspring (CH vs. CC and HH-STZ vs. CC, P < 0.05, Fig 3E). NEFA concentrations were elevated in HFD-fed offspring except in the CH-STZ group (Fig 3F). Maternal obesity significantly heightened NEFA levels in all groups regardless of postnatal diet (P < 0.0001).

### Obesity is associated with impaired renal function and maternal obesity does not exacerbate the effect

**Fig 4:**
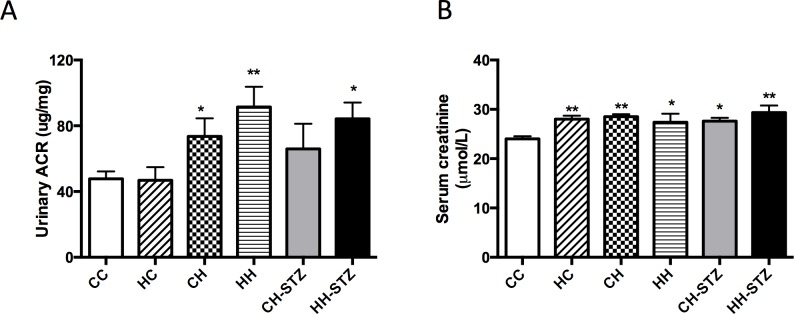
Renal functional changes at Week 32. A. Urinary albumin to creatinine ratio (ACR) at Week 31, B. Serum creatinine concentration at Week 32. Results are expressed as mean ± SEM, N = 9–20. *P< 0.05, **P<0.01 compared to CC.

HFD-induced obesity affected renal function. Specifically, an elevated urinary ACR was evident in almost all groups fed HFD (CH vs. CC, CH-STZ vs. CC, HH-STZ vs. CC, P < 0.05, Fig 4A). In addition, serum creatinine was raised in all groups fed HFD (CH, HH, HH-STZ vs. CC, P < 0.01 and CH-STZ, HH vs. CC, P < 0.05, Fig 4B). Neither STZ-induced injury nor maternal obesity worsened renal function at Week 32.

### Diet-induced obesity and renal structural changes are independent of maternal obesity and STZ injury

**Fig 5:**
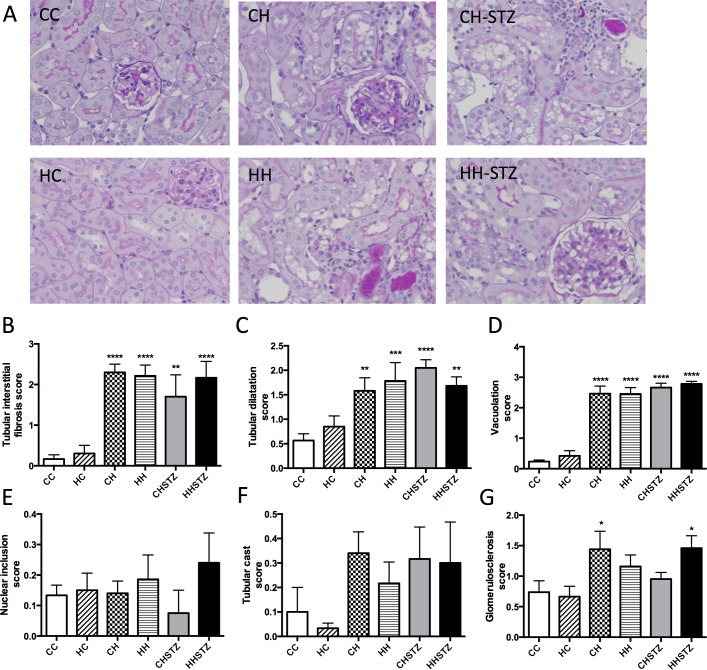
Renal structural changes at Week 32. A. Periodic acid Schiff staining (PAS) representative image at 400x magnification, B. Interstitial fibrosis score, C. Tubular dilatation score, D. Tubular vacuolation score, E. Nuclear inclusion body score, F. Tubular cast score, G. Glomerulosclerosis score. Results are expressed as mean ± SEM, N = 6–7. *P< 0.05, **P<0.01, ***P<0.001, ****P<0.0001 compared to CC.

Obese offspring had marked renal structural changes and in particular, demonstrated renal tubular injury (Fig 5). There was no impact of maternal obesity on renal structural changes beyond diet-induced obesity at Week 32. Tubular interstitial fibrosis is a frequently described renal consequence of diabetes. All HFD-fed groups had evidence of tubular interstitial fibrosis, in particular tubular dilatation, widening of the interstitial space and inflammatory infiltrate (Fig 5A and 5B). There was no effect of maternal obesity on the interstitial fibrosis score with or without HFD feeding.

Further characterisation of tubular injury was performed by assessing tubular dilatation, vacuolation, nuclear inclusion bodies and cast formation. HFD consumption was significantly associated with marked tubular dilatation (CH vs. CC, P < 0.01, HH vs. CC, P < 0.001, CH-STZ vs. CC, P < 0.0001, HH-STZ vs. CC, P < 0.01, Fig 5A and 5C). Moreover, the most consistent and dramatic effect of HFD on renal structure was the appearance of marked vacuolation throughout the tubules (P < 0.0001 for all comparisons, Fig 5A and 5D). There was no difference in nuclear inclusions or casts demonstrated between the groups fed HFD versus chow (Fig 5, 5E and 5F). Renal structure was not influenced by maternal obesity regardless of post-weaning diet.

### Diet-induced obesity has an overwhelming effect on extracellular matrix deposition independent of maternal obesity

**Fig 6:**
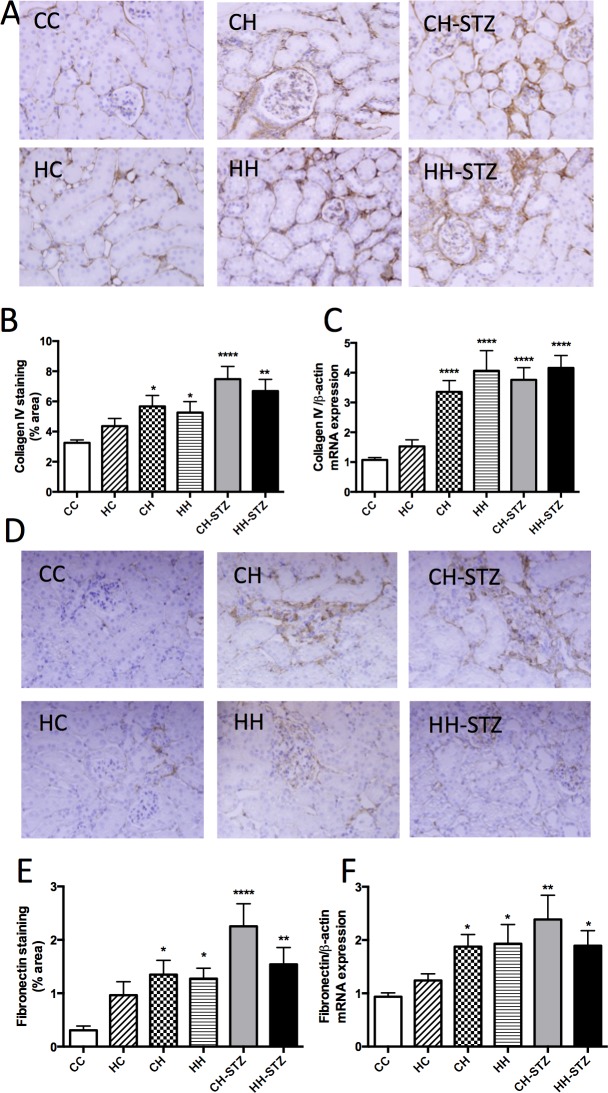
Markers of extracellular matrix deposition at Week 32. A. Collagen IV representative images at 400x magnification, B. Area (%) of collagen IV staining, C. Collagen IV mRNA expression, D. Fibronectin representative images at 400x magnification, E. Area (%) of fibronectin staining, F. Fibronectin mRNA expression. Results are expressed as mean ± SEM, N = 4–6. *P< 0.05, **P<0.01, ***P<0.001, ****P<0.0001 compared to CC.

Extracellular matrix is a hallmark feature of renal fibrosis and is characterised by deposition of major constituents such as collagen IV and fibronectin. There was evidence of collagen IV and fibronectin accumulation in the kidneys of obese mice, evaluated by both immunohistochemistry staining (Fig 6A and 6B) and RT-PCR (Fig 6C). Specifically, collagen IV staining was increased in obese offspring (CH vs. CC, P < 0.05, CH-STZ vs. CC, P < 0.0001, HH-STZ vs. CC, P < 0.01, HH vs. CC, N.S., Fig 6A and 6B). In addition, Collagen IV mRNA expression was increased in all obese offspring (all groups vs. CC, P < 0.0001, Fig 6C). Likewise, fibronectin immunostaining was increased in HFD-fed offspring (CH vs. CC, P < 0.05, HH vs. CC, P < 0.05, CH-STZ vs. CC, P < 0.0001, HH-STZ vs. CC, P < 0.01, Fig 6D and 6E). Fibronectin mRNA expression was increased in all obese offspring (CH vs. CC, P < 0.05, HH vs. CC, P < 0.05, CH-STZ vs. CC, P < 0.01, HH-STZ vs. CC, P < 0.05, Fig 6F). There was no effect of maternal obesity either with or without HFD feeding, or STZ, on either collagen IV or fibronectin.

### Renal inflammation is associated with diet-induced obesity

**Fig 7:**
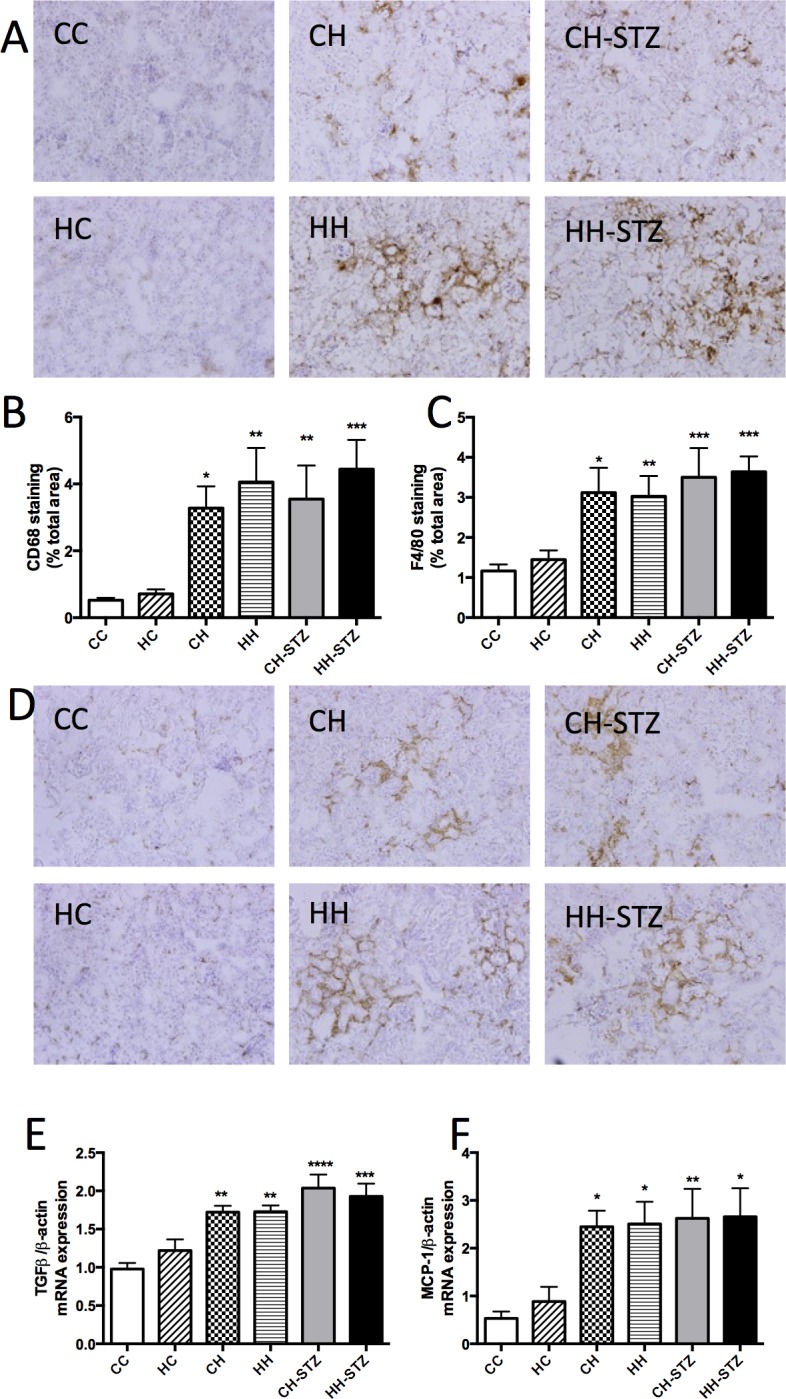
Renal markers of inflammation at Week 32. A. CD68 representative images at 200x magnification, B. Area (%) of CD68 staining, C. Area (%) of F4/80 staining, D. F4/80 representative images at 200x magnification, E. TGF-β mRNA expression, F. MCP-1 mRNA expression. Results are expressed as mean ± SEM, N = 4–6. *P< 0.05, **P<0.01, ***P<0.001, ****P<0.0001 compared to CC.

HFD-induced obesity was associated with increased expression of inflammatory markers CD68 and F4/80 (CH vs. CC, P < 0.05, HH vs. CC, P < 0.01, CH-STZ vs. CC, P < 0.01, HH-STZ vs. CC, P < 0.001, Fig 7A–7D). TGF-β is a pro-inflammatory and pro-fibrotic cytokine and its mRNA expression was increased in all obese groups (CH vs. CC, P < 0.01, HH vs. CC, P < 0.01, CH-STZ vs. CC, P < 0.0001, HH-STZ vs. CC, P < 0.0001, Fig 7E). Similarly the macrophage-mediated pro-inflammatory cytokine MCP-1 was also elevated in the HFD fed groups (CH vs. CC, P < 0.05, HH vs. CC, P < 0.05, CH-STZ vs. CC, P < 0.01, HH-STZ vs. CC, P < 0.05, Fig 7F). In these analyses, there was an overwhelming effect of diet-induced obesity on inflammatory markers and no superimposed effect of maternal obesity was demonstrated.

### Renal oxidative stress is induced by maternal obesity in lean offspring though diet-induced obesity has a stronger impact

**Fig 8:**
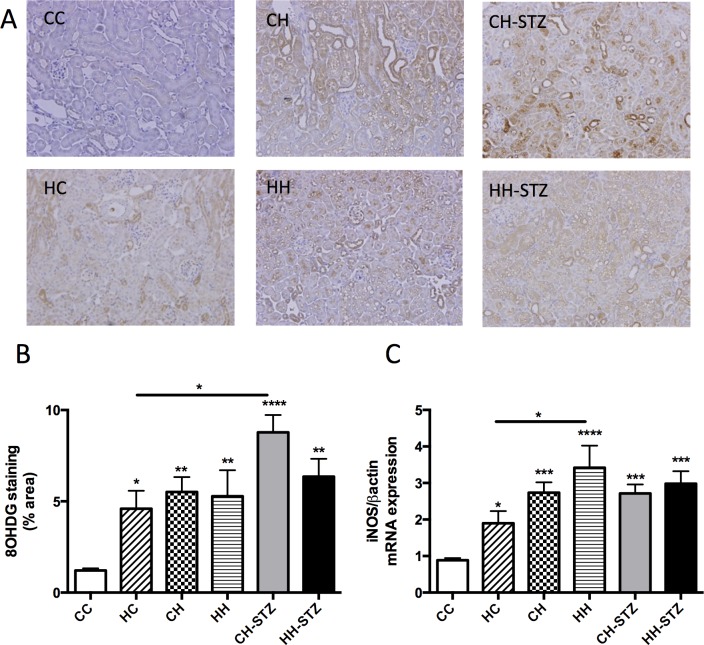
Markers of oxidative stress in the kidney at Week 32. A. 8-OHdg representative images at 200x magnification, B. Area (%) of 8-OHdg staining, C. iNOS mRNA expression. Results are expressed as mean ± SEM, N = 4–6. *P< 0.05, **P<0.01, ***P<0.001, ****P<0.0001 compared to CC.

8-OHdg, a major product of DNA oxidation in the setting of oxidative stress, was measured by immunohistochemistry staining. 8-OHdg expression was increased in obese offspring (CH vs. CC, P < 0.01, HH vs. CC, P < 0.01, CH-STZ vs. CC, P < 0.0001, HH-STZ vs. CC, P < 0.01, Fig 8A and 8B). Though maternal obesity did not exaggerate the effect of HFD on 8-OHdg expression, maternal obesity significantly increased 8-OHdg expression in offspring weaned to normal diet (HC vs. CC, P < 0.05).

The mRNA expression of iNOS was increased in the obese offspring (CH vs. CC, P < 0.001, HH vs. CC, P < 0.0001, CH-STZ vs. CC, P < 0.001, HH-STZ vs. CC, P < 0.001, Fig 8C). Importantly, maternal obesity significantly increased iNOS expression in offspring weaned to a normal diet, though the overwhelming effect of diet-induced obesity mitigated any demonstrable effect of maternal obesity in obese offspring (HC vs. CC, P < 0.05). Indeed, HFD had a more powerful effect on iNOS expression than did maternal obesity (HH vs. HC, P < 0.05).

## Discussion

In this study of maternal and diet-induced obesity, the results demonstrate that diet-induced obesity has multiple detrimental effects on glucose tolerance, metabolic regulation, renal function and structure in C57BL/6 mice. In relation to kidney injury, obesity is associated with functional changes including increased albuminuria and serum creatinine, and structural changes including tubulointerstitial fibrosis, tubular dilatation, and marked vacuolation. In addition, extracellular matrix components, collagen IV and fibronectin, and inflammatory and oxidative stress markers are upregulated in the setting of obesity. The addition of STZ together with HFD had little impact on the renal outcome demonstrated at 24 weeks later, suggesting that one dose of STZ is insufficient to worsen chronic kidney damage induced by dietary obesity. The results suggest that the HFD-induced model of obesity utilising the wild-type C57BL/6 mouse for 32 weeks duration is an excellent model of obesity-related CKD.

Our study also focused on the effect of maternal obesity on offspring kidney health. We found that diet-induced obesity has an overwhelming effect on renal health, overriding any effect of maternal obesity beyond that induced by obesity itself. Nonetheless, in the current murine model we demonstrate a sustained effect of maternal obesity on increasing adiposity, glucose intolerance and lipid concentrations in lean offspring. Moreover, maternal obesity continued to exert an adverse effect on renal function with respect to increased albuminuria and raised serum creatinine in lean offspring. Importantly, markers of oxidative stress were elevated in offspring of obese mothers who weaned to a normal chow diet. This suggests that oxidative stress is likely to be an important mediator of the developmental programming effect of maternal obesity.

Oxidative stress and inflammatory mediators are known to be upregulated in the setting of obesity [17, 18]. In addition, altered cellular programming leading to oxidative stress has previously been recognised as a mechanism underlying the developmental programming effects of maternal obesity on metabolic risk [19, 20]. Increased reactive oxygen species that outweigh anti-oxidant defence mechanisms will lead to cellular injury initiating the inflammatory signalling cascade [21]. Oxidative stress is a key player in initiating and potentiating renal fibrosis and is known to act synergistically with pro-inflammatory cytokines to perpetuate renal damage [22–27]. Van der Heijden and colleagues recently employed a similar mouse model to determine that diet-induced obesity contributes towards renal inflammation and age-dependent amyloidosis [28]. Similar to our results, high fat diet feeding in C57BL/6 mice increased oxidative stress levels in the kidney in the absence of severe pathological changes [29]. Therefore, the findings in this study that maternal obesity and diet-induced obesity up-regulate markers of oxidative stress in the kidney are in keeping with the known role of oxidative stress and inflammation.

A major strength of the present study is the detailed analysis of renal structural changes. Importantly, we observed significant tubulointerstitial fibrosis and tubular dilatation alongside overwhelming evidence of tubular vacuolation in the kidneys of HFD-fed offspring. This finding of tubular vacuolation has been previously described and is associated with lipid accumulation [30–33]. Lipid vacuolation has been observed in proximal tubular cells in mice susceptible to the development of CKD [34]. The HFD-fed offspring in the present study had marked hyperlipidaemia consistent with the abovementioned association between deranged lipid metabolism and vacuolation.

The results of the current study complement our previous studies demonstrating renal effects of maternal obesity until adolescence, related to renal inflammatory, oxidative stress and fibrotic markers [12, 13]. Taken together with the current results, the detrimental effect of maternal obesity on renal health persists to adulthood though diet-induced obesity is a more powerful inducer of renal damage. The addition of STZ together with HFD in the present study was not successful in demonstrating a sustained effect of maternal obesity on renal fibrosis. This may possibly be due to an inadequate dose of STZ, and others have proposed using three doses of STZ with HFD as an alternative [35].

A summary of the cardinal findings of our study can be found in Table 1. We demonstrated that inflammatory and oxidative stress markers are significantly elevated in the kidneys of obese mice and that they have altered functional and structural changes including tubulointerstitial fibrosis and tubular vacuolation. Maternal obesity had a sustained adverse effect on glucose tolerance, lipid metabolism and renal oxidative stress markers; however the harmful effects of diet-induced obesity is even more potent in the long term, and can override the detrimental renal consequences induced by maternal obesity alone.

**Table 1:** Summary of renal effects of maternal obesity, diet-induced obesity and streptozotocin (100mg/kg once) (STZ).

	Maternal obesity	Diet-induced obesity	Diet-induced obesity + STZ	Maternal obesity + diet-induced obesity ± STZ
Kidney function / structural change	↑	↑	↔	↔
Renal fibrotic markers	↑	↑	↔	↔
Inflammatory markers	↑	↑↑	↔	↔
Oxidative stress markers	↑	↑↑	↔	↔

Our results demonstrate that maternal obesity has a sustained effect on renal function and structure independent of diet-induced obesity. However, diet-induced obesity in adulthood has a more potent effect on renal consequences, irrespective of maternal obesity with or without STZ. ↔ denotes no added effect of STZ or maternal obesity beyond that which is demonstrated with diet-induced obesity alone.

## Supporting information

S1 Fig Tubular interstitial fibrosis. Representative images of tubular interstitial fibrosis: A) tubular atrophy, B) tubular dilatation, C) inflammatory infiltrate, D) thickened basement membrane, E) widened interstitial space. Tubular interstitial fibrosis was scored using a scale of 0 to 4: 0 –normal; 1 –involvement of < 10% of the cortex; 2 –involvement of 10–25% of the cortex; 3 –involvement in 25–75% of the cortex, and 4 –extensive damage involving > 75% of the cortex.
(TIFF)

S2 Fig Tubular interstitial injury. Representative images of tubular injury: A) tubular dilatation, B) tubular vacuolation, C) glycogenated nuclei, D) tubular casts). Tubular dilation was scored using a scale of 0 to 4. Tubular vacuolation was scored using a scale of 0 to 3. Glycogenated nuclei were scored using a scale of 0 to 3. Cast appearance was scored as either 0 –absence or 1 –presence.
(TIFF)

S3 Fig Glomerulosclerosis injury. Representative images of glomerulosclerosis scored as A) 0 –no sclerosis, B) 1–25% sclerosis, c) 26–50% sclerosis, D) 51–75% sclerosis, E) > 75% sclerosis.
(TIFF)
