# Supplementary figures and images for: The renal consequences of maternal obesity in offspring are overwhelmed by postnatal high fat diet
Source: PLoS One. 2017 Feb 22;12(2):e0172644. doi: 10.1371/journal.pone.0172644 (PMC5321436; doi:10.1371/journal.pone.0172644)

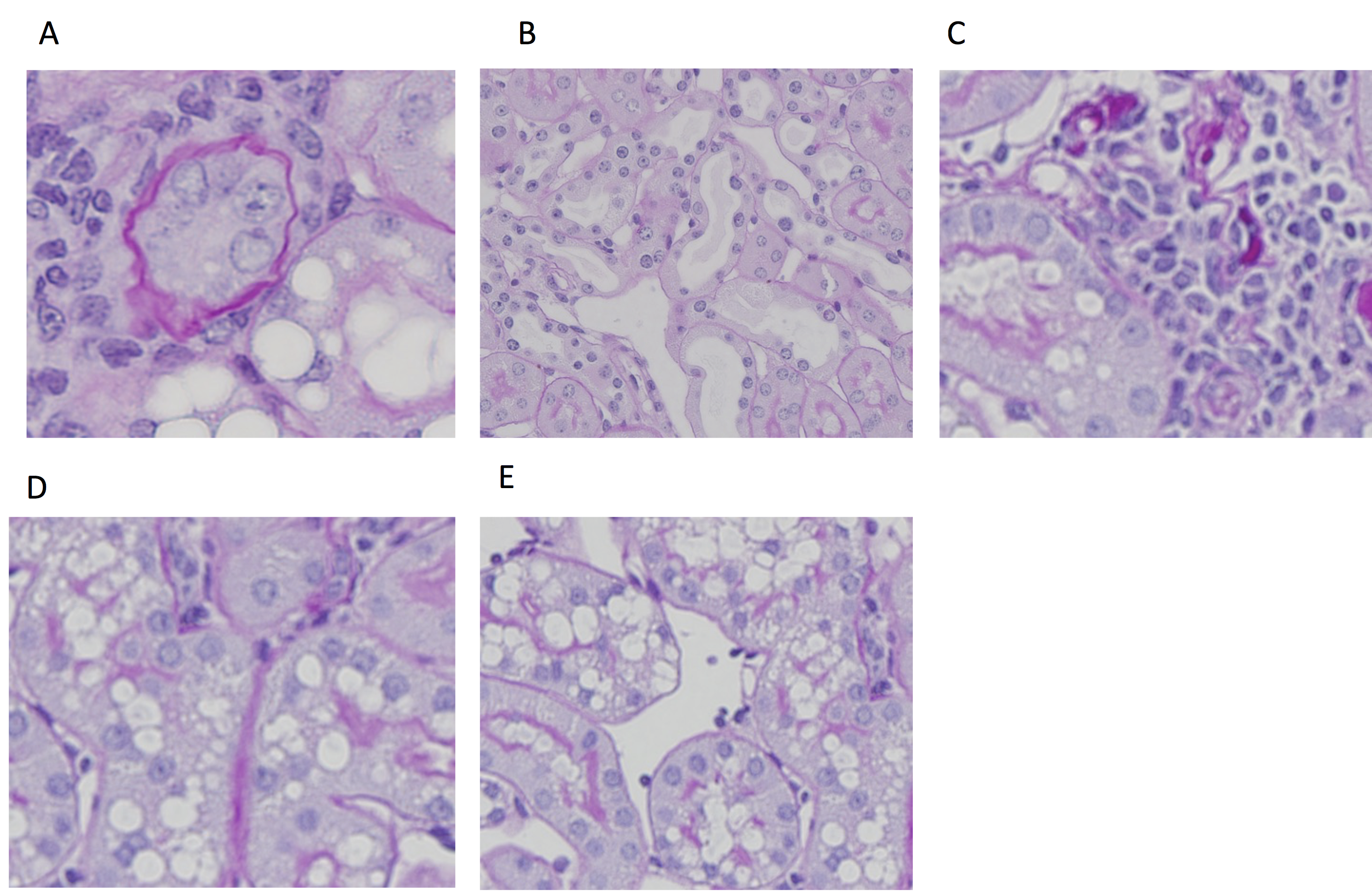

Supplement: S1 Fig — Representative images of tubular interstitial fibrosis: A) tubular atrophy, B) tubular dilatation, C) inflammatory infiltrate, D) thickened basement membrane, E) widened interstitial space. Tubular interstitial fibrosis was scored using a scale of 0 to 4: 0 –normal; 1 –involvement of < 10% of the cortex; 2 –involvement of 10–25% of the cortex; 3 –involvement in 25–75% of the cortex, and 4 –extensive damage involving > 75% of the cortex. (TIFF) [file pone.0172644.s001.tiff]

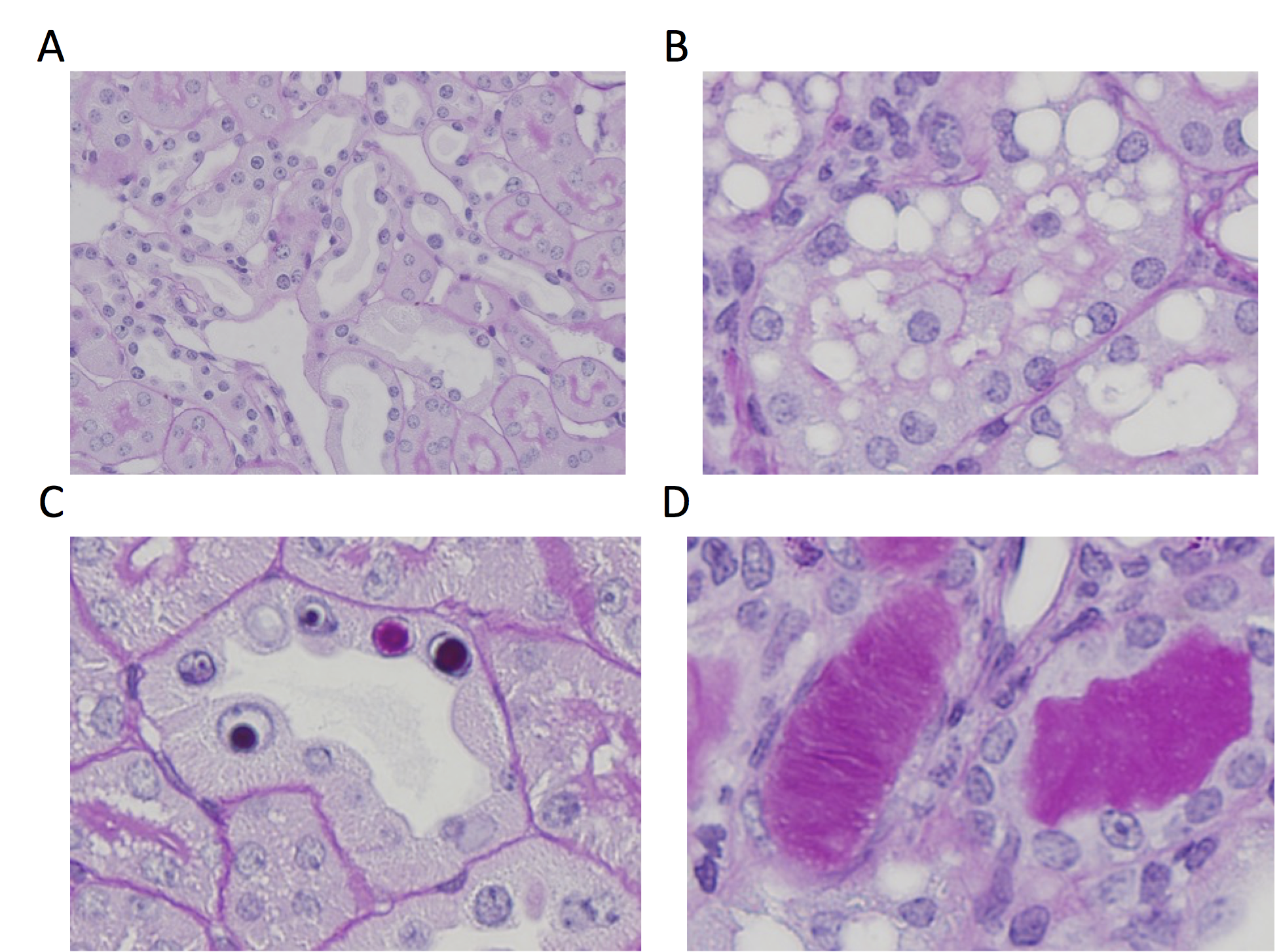

Supplement: S2 Fig — Representative images of tubular injury: A) tubular dilatation, B) tubular vacuolation, C) glycogenated nuclei, D) tubular casts). Tubular dilation was scored using a scale of 0 to 4. Tubular vacuolation was scored using a scale of 0 to 3. Glycogenated nuclei were scored using a scale of 0 to 3. Cast appearance was scored as either 0 –absence or 1 –presence. (TIFF) [file pone.0172644.s002.tiff]

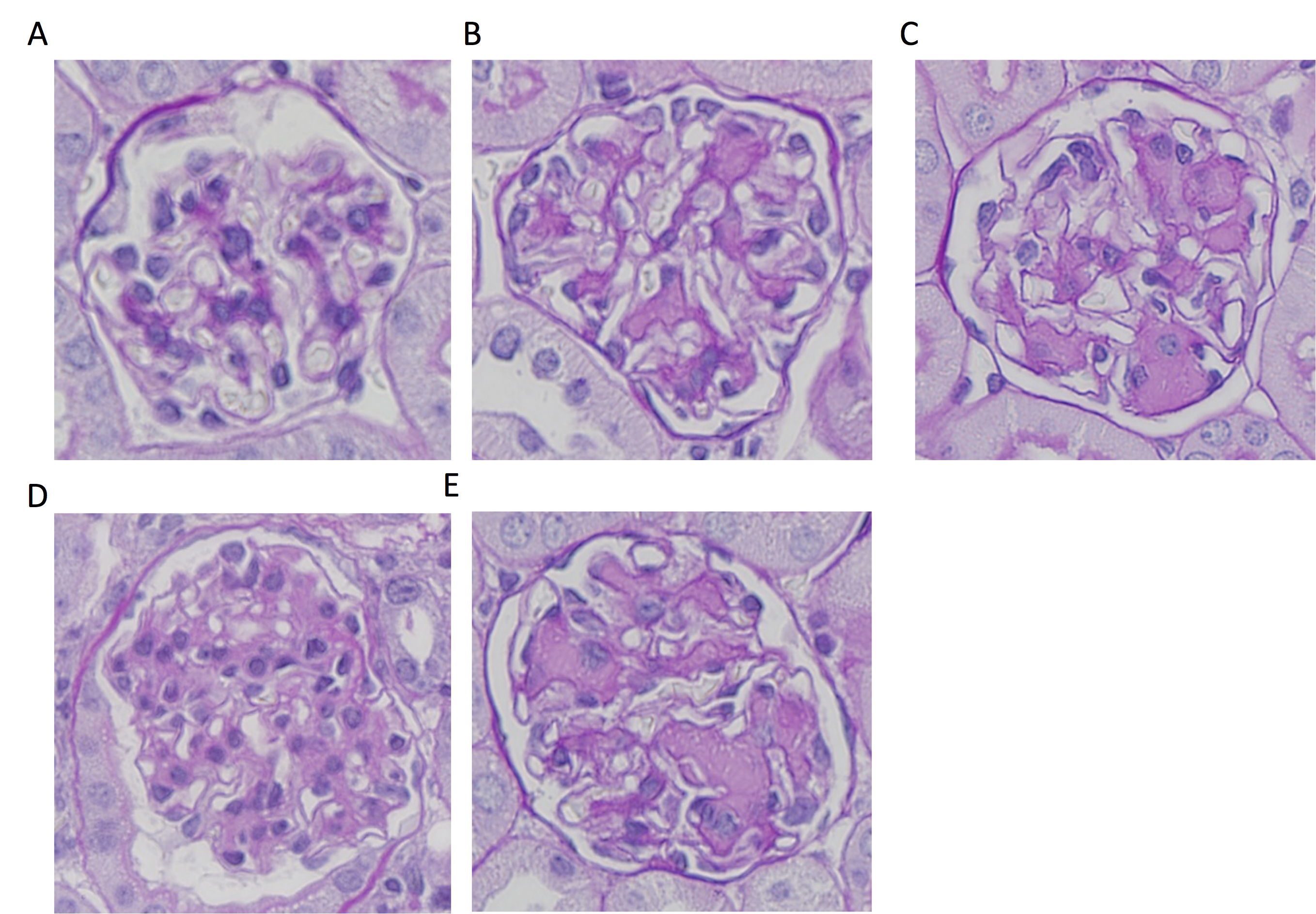

Supplement: S3 Fig — Representative images of glomerulosclerosis scored as A) 0 –no sclerosis, B) 1–25% sclerosis, c) 26–50% sclerosis, D) 51–75% sclerosis, E) > 75% sclerosis. (TIFF) [file pone.0172644.s003.tiff]
